# Preventive Effects of Escitalopram Against Anxiety-Like Depressive Behaviors in Monosodium Glutamate-Teated Rats Subjected to Partial Hepatectomy

**DOI:** 10.3389/fpsyg.2019.02462

**Published:** 2019-11-12

**Authors:** Bin-Bin Zhao, Lin-Lin Chen, Qing-Hua Long, Guang-Jing Xie, Bo Xu, Ze-Fei Li, Ping Wang, Hanmin Li

**Affiliations:** ^1^Hubei University of Chinese Medicine, Wuhan, China; ^2^Hubei Hospital of Traditional Chinese Medicine (Affiliated Hospital of Hubei University of Traditional Chinese Medicine), Hubei University of Chinese Medicine, Wuhan, China

**Keywords:** partial hepatectomy, MSG + PH-treated rats, orbitofrontal cortex, anxiety-like depressive behaviors, Nissl body and neurites, hepatic steatosis, neurotransmitters

## Abstract

The reasons for the relationship between depression and chronic liver disease (CLD) are complex and multifactorial. Further research is needed to decipher the etiology and establish an optimal management approach for depression in patients, including the potential role of non-pharmacological treatments. monosodium glutamate (MSG)-treated rats are more likely to develop anxiogenic- and depressive-like behaviors, which could be related to the dysfunction of serotonergic system. In this study, partial hepatectomy (PH) was performed in MSG-treated rats and the histopathological changes were observed in orbitofrontal cortex (OFC) and liver. The effect of escitalopram, a widely used antidepressant, on neural and liver injury in this model was also examined. The MSG + PH-treated rats displayed decreased distances traveled in total, in center arena, and in the left side of arena in inner open field test (OFT), as compared to saline, saline + PH, and MSG-treated animals. The present study established that PH aggravated anxiety-like depressive behaviors in MSG-treated rats, concordant with damaged Nissl bodies (and neurites), decreased IBA-1 and Sox-2 expression in OFC and neurotransmitter disorder. Escitalopram treatment could alleviate these pathological changes as well as reduce hepatic steatosis and lipid metabolism.

## Introduction

In recent years, mental health conditions, especially depression, have been recognized as a complication of chronic liver disease (CLD). The incidence of depression in patients with CLD is higher than that in general population. Such mental disorders may reduce life quality of patients, worsen clinical outcomes, reduce compliance and increase mortality of liver disease treatment (Popovic et al., 2015). Approximately 10–20% of hepatitis B virus (HBV) carriers become chronically infected, which can lead to fibrosis and even cirrhosis. Given the high prevalence of HBV infection in China and the high relapse rate of viral hepatitis, CLD patients typically experience panic, depression, and anxiety. Furthermore, participants with comorbid major depression and liver disease showed higher rates of lifetime suicide attempts (33.2%) than general population [13.7%; odds ratio (OR): 3.1; confidence interval (CI): 1.3–7.6] (Le Strat et al., 2015). A meta-analysis also found that psychological distress was associated with liver disease mortality (Russ et al., 2015). Psychological factors that increase vulnerability to the temptation to use alternative medicines, such as herbs and plant preparations, have been revealed to be important for understanding toxic liver injury (Suh et al., 2013). In the presence of hepatic encephalopathy (HE) in patient with depressive symptoms, HE-directed therapies should be attempted before antidepressant drugs (Mullish et al., 2014; Telles-Correia et al., 2015). Nevertheless, the reasons for the relationship between depression and CLD are complex and multifactorial. Further research is needed to decipher the etiology and establish an optimal management approach for depression in these patients, including the potential role of non-pharmacological treatments.

The monosodium glutamate (MSG)-rat model is a well-characterized animal obesity model that is used to study many metabolic syndromes, such as lipid metabolism and insulin resistance (Dolnikoff et al., 2001; Caetano et al., 2017). Our earlier studies established a compromised liver regeneration model by applying the partial hepatectomy (PH) operation in MSG-treated rats. After undergoing PH, the MSG-induced animals displayed disorders of the neuroendocrine-immune network (NEIN) (Dolnikoff et al., 2001; Caetano et al., 2017) as well as liver injury (Li, 2014). The numbers of hypothalamic neurons were significantly reduced and increased apoptosis was observed. In addition, liver regeneration was suppressed in the MSG-rats after PH as compared to saline-treated rats. Recently, it was shown that MSG-treated rats are more likely to develop anxiogenic- and depressive-like behaviors, which could be related to the dysfunction of serotonergic system (Quines et al., 2014, 2015). Magnetic resonance imaging (MRI) studies revealed that increased functional connectivity of the lateral orbitofrontal cortex (OFC) Brodmann area 47/12 is related to depression. It is reported that changes in cortical connectivity within the OFC are critical to depression (Gao et al., 2015). In the present study, we performed PH in MSG-treated rats and observed the histopathological changes in OFC and liver. The effect of escitalopram, a widely used antidepressant, on neural and liver injury in this model was also examined.

## Materials and Methods

### Reagents

Dopamine (DA), 5-hydroxytryptamine (5-HT), norepinephrine (NE), acetylcholine (Ach), epinephrine (Epi), glutamate (Glu), gamma-aminobutyric acid (GABA), 5-hydroxyindoleacetic acid (5HIAA), and 3,4-dihydroxybenzylamine hydrobromide (IS) were purchased from Sigma-Aldrich (St. Louis, MO, United States). Escitalopram was obtained from Forest Pharmaceuticals, Inc. (New York, NY, United States). Escitalopram was prepared as a 0.105 mg/ml stock solution in saline.

### Establishment of MSG + PH Rat Model and Escitalopram Administration

Neonatal male Wistar rats were purchased from the Hubei Experiment Animal Research Center. Twenty-four rats were given subcutaneous injection of the MSG solution (in normal saline) at a dosage of 4 mg/g body weight (bw), and 16 rats were injected with the same volume of vehicle (saline) on days 2, 4, 6, 8, and 10 after birth. The pups were weaned on day 8 and caged at 6 weeks old in five groups, receiving saline or escitalopram via gastrogavage for 2 weeks. PH was performed during week 8 by excision of the left and median hepatic lobes (occupying about 68% of whole liver) according to the Solt-Farber method under ether anesthesia (Zhao et al., 2015). The groups (*n* = 8/group) received the following treatments: (1) saline daily via gastrogavage; (2) saline + PH; (3) MSG injection + saline; (4) MSG + PH; and (5) MSG +PH + escitalopram gastrogavage to the endpoint of the experiment (1 ml/100 g bw of the 0.105 mg/ml stock solution daily, corresponding to 10 mg/60 kg bw daily in humans). The rats were maintained in an air-conditioned (temperature 24 ± 1°C; 55 ± 5% relative humidity) animal room with controlled lighting (12 h light, 12 h darkness). They were provided with a commercial diet and water. On day 8 after PH, the rats were subjected to an open field test (OFT) before being sacrificed by CO_2_ as phyxiation followed by cervical dislocation. The blood, liver, and OFC tissues were collected and then snap frozen or fixed in formalin. All animal handling and procedures were approved by the Institute of Animal Care and Use Committee of the Hubei University of Traditional Chinese Medicine.

### Open Field Test

The inner OFT was conducted in an open arena (l, w, h: 50 cm × 50 cm × 40 cm) with the bottom and sides made of Plexiglas covered with black, nonreflecting material. The arena was placed in a quiet room. On day 8 after PH, the rats were individually placed in the center of the apparatus and left to move freely during a 3 min period with their movements being automatically recorded using a camera connected to a computer (ZH-ZFT, Zhenghua Biological Instrument Equipment Co., Ltd., Huaibei, China). The total distance moved (cm) and total distance traveled (cm) from the center area were analyzed.

### Histological and Immunohistochemical Staining

After dewaxing and hydration of paraffin sections, the liver and cortex tissues were stained with hematoxylin and eosin (H&E). The sections of frozen liver tissue were stained with Oil Red O (ORO). Nissl staining was performed on the OFC samples for Nissl body observation. The OFC was also stained with anti-brain-derived neurotrophic factor (BDNF, sc-33904), anti-Sox-2 (sc-365964), and anti-ionized calcium-binding adapter molecule 1 (IBA-1, sc-32725; Santa Cruz Biotechnology, Santa Cruz, CA, United States). Five fields on each slide were randomly selected, viewed under a fluorescence microscope (Nikon TE2000-U, Nikon, Japan), and analyzed using Image Pro-Plus 6.0 software (Media Cybernetics, Silver Spring, MD, United States). The minimal pixel number was set at 50 pixels. The average and cumulative optical density values, average area, and average diameter were analyzed. StreptAvidin Biotin Complex kits were purchased from Boster Biological Technology Co., Ltd. (Wuhan, China).

### Lipid Metabolism Analysis

The serum levels of total cholesterol (TC), triglycerides (TG), low-density lipoprotein cholesterol (LDL), and high-density lipoprotein cholesterol (HDL)were measured using commercially available enzyme-linked immunosorbent assay (ELISA) kits according to the manufacturer’s instructions (Yuanye Bio-Technology Co., Ltd., Shanghai, China).

### Neurotransmitter Levels

Neurotransmitter levels in the OFC were determined by a previously reported method with minor modification (Huang et al., 2014). Briefly, about 40 mg of OFC tissue was homogenized in a 1.5 ml Eppendorf tube after addition of 400 μl ice cold methanol (0.1% formic acid) and 10 μl IS (10 μg/ml, methanol). The homogenate was vortex-mixed for 1 min and then centrifuged at 18,000 × *g* for 10 min at 4°C. The supernatant was transferred and evaporated to dryness under a nitrogen stream. The dry residue was reconstituted in 100 μl of initial mobile phase (0.1% formic acidin water/acetonitrile, 98:2, v/v), and a 10-μl aliquot was injected into the liquid chromatography-tandem mass spectrometry (LC-MS/MS) system for analysis. The sample was run on an Agilent 1290 Infinity series connected to an Agilent 6420 triple quadrupole mass spectrometer equipped with an electrospray ionization (ESI) ion source (Agilent Technologies, Santa Clara, CA, United States). The analytes were separated on a Waters BEH C_18_ column (2.1 mm × 100 mm, 1.7 μm, Waters, Milford, MA, United States) at 30°C. The mobile phase consisting of 0.1% formic acid in water (Solvent A) and acetonitrile (Solvent B) was used with a gradient elution: 0–4 min, 2% B; 6 min, 80% B; 8–10 min, 90% B at a flow rate of 0.3 mL/min. The ESI-MS/MS conditions were set as following: gas temperature 350°C, gas flow 10 L/min, capillary 4000 V, and nebulizer pressure 30 psi. MS acquisition of NE, 5-HT, DA, Glu, GABA, Ach, Epi, and 5-HIAA spectra was performed in electrospray positive ionization multiple reaction monitoring (MRM) mode.

### Statistical Analysis

Data are expressed as mean ± standard deviation (SD). Statistical analyses were performed using SPSS software (version 19.0; IBM, Armonk, NY, United States). Inter-group comparison was performed by one-way analysis of variance (ANOVA). *P* < 0.05 was considered statistically significant.

## Results

### Anxiety-Like Depressive Behaviors in Model Rats

**FIGURE 1:**
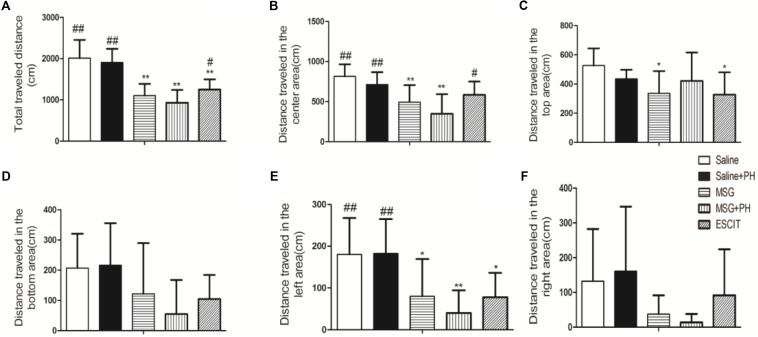
Effect of escitalopram on the anxiety-like depressive behaviors in MSG + PH-treated neonatal rats. **(A)** Total traveled distance in the open arena; **(B)** distance traveled in the center area of the open arena; **(C)** distance traveled in the top area; **(D)** distance traveled in the bottom area; **(E)** distance traveled in the left area; and **(F)** distance traveled in the right area; ^∗^*P* < 0.05, compared to the saline group; ^∗∗^*P* < 0.01, compared to the saline group. ^#^*P* < 0.05, compared to the MSG + PH group; ^##^*P* < 0.01, compared to the MSG + PH group. *n* = 8 per group.

We first examined the effect of PH and escitalopram on the anxiety-like behaviors of neonatal rats. As shown in Figure 1, the rats treated with saline and no PH traveled a total distance of 2014.56 ± 441.06 cm in 3 min in the OFT experiments. The distances traveled in the center and the left side of open arena were 814.93 ± 150.30 and 180.46 ± 87.24 cm, respectively. The saline + PH-treated animals traveled a total distance of 1908.78 ± 330.96 cm, 712.74 ± 154.19 cm in the center, and 182.65 ± 82.59 cm on the left side (*P* > 0.05 vs. saline-treated rats). The MSG-treated rats without PH traveled a total distance of 1103.14 ± 285.99 cm, 494.83 ± 210.80 cm in the center, and 80.05 ± 89.23 cm on the left, and these distances were significantly shorter than those traveled by saline-treated animals without PH (*P* < 0.01 or *P* < 0.05), suggesting that MSG treatment induced anxiety-like depressive behaviors of neonatal rats. In addition, the MSG-induced rats with PH showed even further decreases (but not significantly) in the total distance (932.05 ± 306.65 cm) as well as the distance traveled in the center of the arena (349.83 ± 243.47 cm) and on the left (40.23 ± 51.11 cm) compared to MSG-treated rats without PH (but all *P* < 0.01 vs. the saline group). These results confirmed the presence of anxiety-like depressive behaviors in MSG + PH-treated animals. The administration of escitalopram to MSG + PH-treated animals resulted insignificantly increased distances traveled in total (1252.42 ± 245.52 cm; *P* < 0.05), in the center (584.57 ± 165.68 cm; *P* < 0.05), and on the left side (77.93 ± 58.15 cm; *P* > 0.05) compared to those of the MSG + PH-treated animals. Therefore, we successfully established a liver regenerative model in MSG-treated neonatal rats with anxiety-like depressive behaviors, and escitalopram could partially alleviate the anxiety-like depressive behaviors in this model.

### Histopathological Analyses of OFC

**FIGURE 2:**
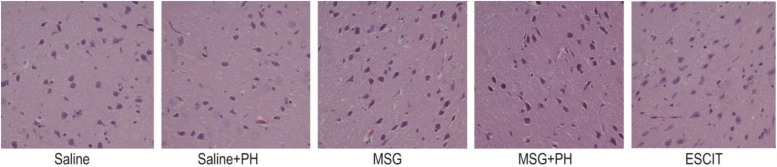
Effect of escitalopram on OFC histological features in MSG + PH-treated neonatal rats. Sections were stained using H&E. Magnification, 400×.

Representative H&E staining of the OFC is shown in Figure 2. The neurons of the saline-treated rats had an integral morphology and structure with normal synapses. Saline-treated rats subjected to PH had relatively integral neurons, yet some of the neurons exhibited shrunken soma and reduced synapses. In contrast, the neurons in the OFC of the MSG-treated animals were largely damaged, exhibiting a reduced size in the neuronal soma and disappearance of the synapses. These effects were aggravated in the MSG + PH-treated rats. Escitalopram administration partially rescued the cortical neurons, as evidenced by an increased volume of synapses and recovery of neuron morphology.

**FIGURE 3:**
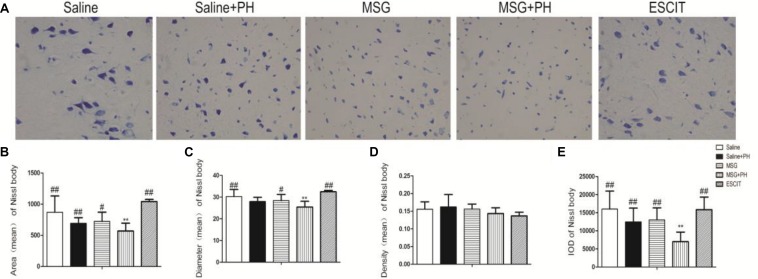
Effect of escitalopram on the Nissl body morphology and neurite outgrowth in neonatal rats. **(A)** Nissl body staining (400×); **(B)** mean area; **(C)** mean diameter; **(D)** mean density; and **(E)** cumulative IOD. ^∗^*P* < 0.05 compared to the saline group; ^∗∗^*P* < 0.01 compared to the saline group. ^#^*P* < 0.05 compared to the MSG + PH group; ^##^*P* < 0.01 compared to the MSG + PH group. *n* = 4∼8 per group, and five fields were recorded from each specimen.

We next sought to investigate the protective effect of escitalopram on the cortical Nissl bodies and neurite outgrowth (Figure 3). As shown in Figure 3A, the saline-treated animals showed integral Nissl bodies in the OFC with long and thick neurites. The mean area was 869.82 ± 261.16, the mean diameter of Nissl bodies was 30.26 ± 3.31, and the IOD of stained tissue was 16018.16 ± 4968.46. PH decreased the volume of Nissl bodies (mean area 691.26 ± 90.89; IOD 12467.89 ± 3811.55; *P* > 0.05). The neurons exhibited abnormal morphology, including shortening and thinning of the neurites (Figures 3A–C). This was also significant in the OFC of MSG-treated rats with even weaker Nissl staining and the disappearance of some synapses. There were no significant differences between the MSG group and the saline group. In contrast, MSG + PH-treated animals showed significant differences in these parameters as compared to MSG-treated rats, indicating that PH significantly aggravated the neural injury in MSG-treated rats. PH in MSG-treated rats led to significantly decreased staining of the neurons (Figure 3) and the disappearance of almost all synapses, with a mean area of 586.59 ± 125.74, mean diameter of the Nissl bodies of 25.41 ± 2.66, and IOD of 6981.17 ± 2667.62. In contrast, we observed that administration of escitalopram largely protected the integrity of the Nissl bodies and the morphology of neurites (mean area of 1080.89 ± 35.22, mean diameter of the Nissl bodies of 32.45 ± 0.62, and IOD of 15849.69 ± 3466.60, all ^∗^*P* < 0.01 compared to the MSG + PH group). These results demonstrated that escitalopram could attenuate the neural injury in this model.

### IHC of OFC

**FIGURE 4:**
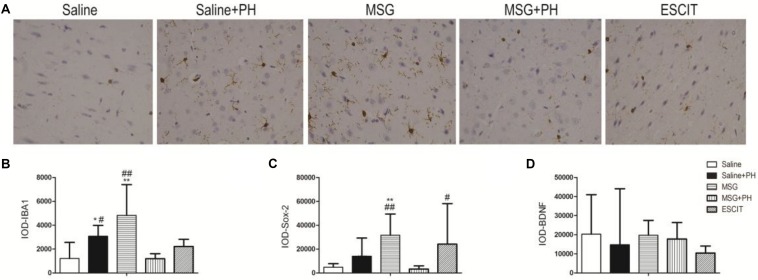
Effect of escitalopram on the expression of markers for microglia and NSC as well as neurotrophic factors in the OFC of neonatal rats by immunohistochemistry. **(A)** OFC sections were stained using anti-IBA-1 antibody (400×). **(B)** IOD for IBA-1; **(C)** IOD for Sox-2; **(D)** IOD for BDNF. ^∗^*P* < 0.05 compared to the saline group; ^∗∗^*P* < 0.01 compared to the saline group. ^#^*P* < 0.05 compared to the MSG + PH group; ^##^*P* < 0.01 compared to the MSG + PH group. *n* = 4∼8 per group, and five fields of each specimen were recorded.

We next examined the expression of IBA-1 (microglia marker), Sox-2 [neural stem cell (NSC) marker], and neurotrophic factors. As shown in Figure 4, the IOD for IBA-1 in the saline + PH group and MSG group were 3073.63 ± 912.00 and 4820.61 ± 2582.22, respectively, and these values were significantly higher than that in the saline group (1208.58 ± 1348.46; *P* < 0.01 or *P* < 0.05). The MSG + PH-treated rats displayed lower IBA-1 expression (1196.84 ± 418.61; *P* < 0.01) than the two groups that received only one individual treatment. The IOD for Sox-2 in the MSG group was 31666.59 ± 17705.65, which was significantly higher than that in the saline group (4812.43 ± 2985.14; *P* < 0.01). The MSG + PH-treated rats displayed lower expression of Sox-2 (3263.81 ± 2654.46; *P* < 0.01) than those treated with MSG only (31666.59 ± 17705.65, *P* < 0.01). There was no significant difference in BDNF expression among all the groups related to the saline-treated control group. Escitalopram treatment enhanced Sox-2 expression (24128.59 ± 34003.34; *P* < 0.01) compared to that in the MSG + PH group. These results demonstrated that MSG and PH led to decreased expression of microglial and NSC markers, but escitalopram treatment could partially restore the expression levels of these proteins.

### Liver Histological Analysis

**FIGURE 5:**
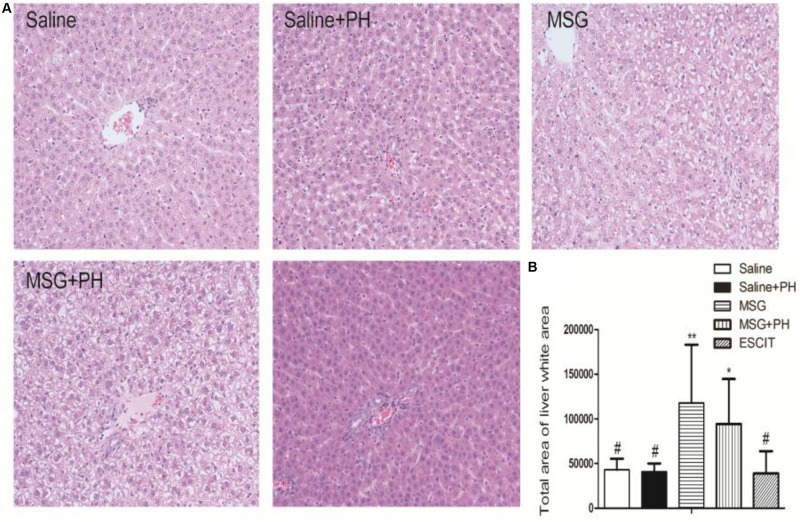
Effect of escitalopram on liver histology in MSG + PH-treated neonatal rats. **(A)** Liver sections were stained using H&E (200×). **(B)** Statistical analysis of the liver white area for different groups of animals. ^∗^*P* < 0.05 compared to the saline group; ^∗∗^*P* < 0.01 compared to the saline group. ^#^*P* < 0.05 compared to the MSG + PH group. *n* = 8 per group.

Representative images of liver histology using H&E staining are shown in Figure 5. Normal liver histology with typical lobular architecture was observed in saline-treated rats. Eight days after PH, the liver of the neonatal rats exhibited a similar initial architecture with only a few small fat vacuoles. In contrast, the liver lobes in the MSG group displayed diminished borderlines with loosening cytoplasmic structures in the hepatocytes. There were increased numbers of hepatic vacuoles and liver cells, especially those located centrally in the lobuli, which displayed considerable swelling with vacuolization and a balloon*-*like appearance similar to adipocytes. MSG + PH-treated rats exhibited more disturbances in the structure of the liver, as evidenced by the disappearance of borderlines and more balloon*-*like hepatocytes and vacuolization. The administration of escitalopram attenuated the changes in liver architecture, reducing vacuolization of the liver, although a few balloon*-*like hepatocytes were still observed.

The white areas were further analyzed in the H&E-stained slides. The unknown tiny particles were discarded by setting the minimum pixel value to 50 pixels, and the white portal areas were excluded by setting the maximum pixel value to 5000 pixels. As shown in Figure 5, the MSG + PH group had a significantly larger white area in the liver architecture, at 94256.37 ± 50510.38, compared with the saline group (43158.82 ± 12323.81; *P* < 0.05) and the saline + PH group (40799.50 ± 9360.42; *P* < 0.05). Escitalopram treatment significantly reduced the white area (38972.78 ± 24694.60; *P* < 0.05). These data suggest that the escitalopram was effective at alleviating liver injury and enhancing liver regeneration.

### Analysis of Liver Steatosis

**FIGURE 6:**
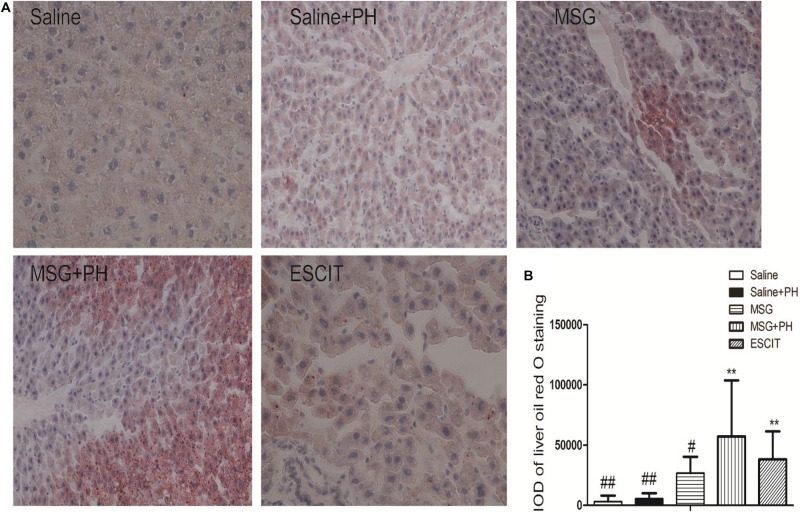
Effect of escitalopram on lipid deposition in the liver of MSG + PH-treated neonatal rats. **(A)** Liver tissues were stained with ORO (400×). **(B)** Statistical analysis of the difference between different groups. ^∗∗^*P* < 0.01 compared to the saline group. *n* = 8 per group. ^#^*P* < 0.05 compared to the MSG + PH group; ^##^*P* < 0.01 compared to the MSG + PH group. *n* = 8 per group.

Hepatic steatosis was analyzed as follow in different groups. Frozen liver tissues were sectioned and stained with ORO solution. The saline group had an IOD for lipid deposition of 3214.23 ± 4843.85, and the saline + PH group had an IOD of 5559.96 ± 4475.62. The MSG group had an IOD of 26852.50 ± 13472.09. Compared with lipid deposition in the MSG + PH group (IOD of 57169.03 ± 46660.75), escitalopram administration during the recovery period after PH significantly alleviated liver steatosis (38179.23 ± 23306.88; Figure 6). These results demonstrated that MSG-treated rats had significant liver steatosis and PH further increased the level of steatosis. However, escitalopram treatment could protect against PH- and MSG-induced hepatic steatosis.

### Lipid and Insulin Profiles

**FIGURE 7:**
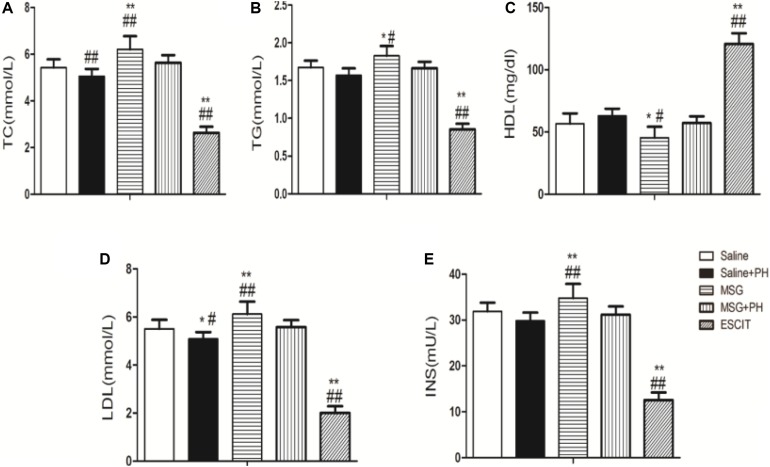
Effect of escitalopram on serum levels of lipids and insulin in MSG + PH-treated neonatal rats. **(A)** TC; **(B)** TG; **(C)** HDL; **(D)** LDL; and **(E)** insulin levels.^∗^*P* < 0.05 compared to the saline group; ^∗∗^*P* < 0.01 compared to the saline group. *n* = 8 per group. ^#^*P* < 0.05 compared to the MSG + PH group; ^##^*P* < 0.01 compared to the MSG + PH group. *n* = 8 per group.

The serum lipid and insulin profiles were examined in the different groups. As shown in Figure 7, MSG treatment increased the serum levels of TC (6.21 ± 0.57 mmol/L), TG (1.83 ± 0.13 mmol/L), LDL (6.12 ± 0.52 mmol/L), and insulin (34.79 ± 3.09 mU/L) compared to the levels following saline treatment (TC: 5.42 ± 0.36 mmol/L, *P* < 0.01; TG: 56.68 ± 8.21 mmol/L, *P* < 0.01; LDL: 5.51 ± 0.38 mmol/L, *P* < 0.01; insulin: 31.87 ± 1.92 mU/L, *P* < 0.01). MSG + PH-treated rats had decreased serum levels of TC (5.63 ± 0.33 mmol/L; *P* < 0.01), TG (1.66 ± 0.08 mmol/L, *P* < 0.05), LDL (5.57 ± 0.29 mmol/L, *P* < 0.05), and insulin (31.18 ± 1.79 mU/L, *P* < 0.01) than those treated with MSG only. Interestingly, escitalopram treatment further decreased the serum levels of TC (2.63 ± 0.26 mmol/L), TG (0.85 ± 0.07 mmol/L), LDL (2.01 ± 0.28 mmol/L), and insulin (12.60 ± 1.62 mU/L) (*P* < 0.05 or < 0.01). Opposite changes were seen for the serum level of HDL in the saline (56.68 ± 8.21 mg/dl), MSG (45.44 ± 8.75 mg/dl; *P* < 0.01 vs. saline group), MSG + PH (57.14 ± 5.43 mg/dl; *P* < 0.05 vs. MSG group), and escitalopram-treated groups (120.78 ± 8.50 mg/dl; *P* < 0.01 vs. MSG + PH group). Therefore, the MSG-treated animals had abnormal metabolism and PH could aggravate the lipid deposition. In contrast, escitalopram could improve lipid metabolism. Inconsistently, there were lower serum levels of TC, TG, LDL, and insulin in the saline + PH and MSG + PH groups, as compared to the saline and MSG groups, indicative of compromised liver function in rats that received PH prior to complete liver regeneration.

### OFC Neurotransmitter Profile

**FIGURE 8:**
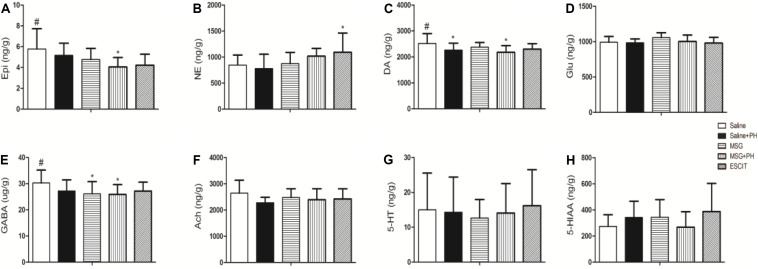
Effect of escitalopram on OFC levels of neurotransmitters in MSG + PH-treated neonatal rats. **(A)** Epi; **(B)** NE; **(C)** DA; **(D)** Glu; **(E)** GABA; **(F)** Ach; **(G)** 5-HT; and **(H)** 5-HIAA levels in the OFC. ^∗^*P* < 0.05 compared to the saline group. ^#^*P* < 0.05 compared to the MSG + PH group. *n* = 4∼8 per group.

Lastly, the OFC neurotransmitter profile was investigated in the different treatment groups (Figure 8). The Epi level in the OFC of the MSG + PH group was 4.05 ± 0.90 ng/g, which was significantly lower than that in the saline group (5.78 ± 1.94 ng/g; *P* < 0.05). Compared to NE expression in the saline group, escitalopram treatment significantly increased the expression of NE (847.74 ± 191.77 vs. 1094.24 ± 367.91 ng/g; *P* < 0.05). The DA level in the OFC of the MSG + PH group was 2178.28 ± 257.76 ng/g, which was significantly lower than that in the saline group (2517.95 ± 381.10; *P* < 0.05). The GABA levels reduced in both MSG (26.11 ± 4.67 ng/g) and MSG + PH group (25.89 ± 3.77 ng/g), which significantly lower than that in the saline group (30.24 ± 4.95 ng/g). The OFC neurotransmitter profile showed that levels of a variety of neurotransmitters, including DA, GABA, and 5-HT, were decreased in rats treated with MSG and subjected to PH compared to those in MSG-treated animals not subjected to PH. Moreover, these changes could be alleviated by escitalopram treatment.

## Discussion

In the present study, we established a compromised liver regeneration model by performing PH in MSG-treated neonatal rats. The animals displayed anxiety-like depressive behaviors after MSG treatment, which was aggravated by PH. Transient lipid accumulation within hepatocytes preceding the peak proliferative phase is a characteristic feature of the regenerating liver (Kohjima et al., 2013). As evidenced by the liver HE and ORO staining, MSG-treated rats exhibited hepatic steatosis, and PH aggravated the degree of steatosis. However, administration of the antidepressant escitalopram could not only alleviate this anxiety-like depressive behaviors, but also reduce hepatic steatosis and improve liver function.

The neurobiological mechanisms of depression are complex, and the relationship between depression and CLD is multifactorial. Some factors, such as life stress and endocrine abnormalities, are thought to cause depression. In addition, genetic and environmental factors play important roles in the onset of depression in relation to epigenetics (Nabeshima and Kim, 2013). It involves signaling cascades within a complicated network, including the monoamine neurotransmitter system, the neuroendocrine system, neurotrophic factors, adult nerve regeneration, the neural immune system, and epigenetic modification. The hypothalamic-pituitary-adrenal axis (HPA) and the production of proinflammatory cytokines have also been implicated in the pathophysiology of depression (Leonard, 2006; Stepanichev et al., 2014; Jeon and Kim, 2016). Our earlier work revealed that, compared to the liver of normal rats subjected to PH, decreased mRNA and protein expression levels of transforming growth factor alpha (TGF-α) and epidermal growth factor receptor (EGFR) were observed in regenerated liver tissues. However, the expression of TGF-β1 and TGF-β type I receptors (TGFBRI and TGFBRII) were upregulated in the arcuate nucleus (ARN) of MSG-induced rats (Li, 2014). These cytokines would contribute to the compromised liver regeneration and disturbance of the NEIN. We have identified that the traditional Chinese medicine (TCM) formulation, Zuo Gui Wan (ZGW), can regulate the NEIN and consequently modulate liver regeneration (Li et al., 2005, 2006). In contrast, ZGW also can significantly increase the expression of TGF-α and EGFR, while decreasing the expression of TGFBRI and TGFBRII (Li et al., 2004; Li, 2014, 2017).

Escitalopram is an antidepressant of the selective serotonin reuptake inhibitor (SSRI) class and is mainly used to treat major depressive disorder or generalized anxiety disorder. Escitalopram was shown to have an antioxidant effect associated with an increase in GABA levels in frontal cortices and nucleus accumbens homogenates from rats exposed to chronic mild stress (Shalaby and Kamal, 2009). Our results revealed that MSG treatment decreased GABA levels and MSG + PH treatment decreased both DA and GABA levels in the OFC of rats. Administration of escitalopram alleviated the disorder of the neurotransmitter profile in the OFC by increasing the level of NE. Escitalopram is also known to reduce inflammation in depression. Stress-related increases in proinflammatory cytokines may underlie the oxidative and nitrosative brain damage and the impairment of the 5-HT system (Maes, 2008). In a rat model of post-cardiac infarct depression, the antidepressant effect of escitalopram was shown to reduce circulating pro-inflammatory cytokines (tumor necrosis factor alpha [TNF-α], interleukin-1β, and prostaglandin E2) and improve depressive behavior without affecting sleep (Bah et al., 2011). A higher level of TNF-α might predict a non-response to treatment with escitalopram (Eller et al., 2008). As the cytokines and neurotransmitters affect neurogenesis in brain regions involved in depression and are functionally interconnected, the alteration of these profiles by MSG and PH might underlie the decreased expression of microglial and NSC markers (Lauterbach, 2016). The present study revealed that escitalopram could partially restore survival and proliferation of NSC while reducing microglial activation.

Escitalopram and its prodrug citalopram are extensively metabolized in the liver, mainly via the cytochrome P450 system (CYP 3A4, 2D6, and 2C19). Genetic polymorphisms of cytochrome P450 enzymes have been shown to influence the metabolism of escitalopram as well as treatment response (Tsai et al., 2010). As such, the maximum daily dose of escitalopram for patients with hepatic insufficiency is 10 mg. In addition, the efficacy of escitalopram in mild or moderate cases of depression has been disputed and may be outweighed by its side effects. To date, no serious liver injury has been reported with the use of escitalopram. We showed that PH led to liver regeneration in MSG-treated rats; however, the regeneration was compromised as evidenced by the greater hepatic steatosis compared with that in saline-treated animals subjected to PH. On the other hand, PH aggravated the histopathological changes in MSG rats. Depression and metabolic syndrome may have a common neuroendocrine and immune basis (Hess et al., 2004; Ghanei Gheshlagh et al., 2016; Song and Kim, 2016; Ohmori et al., 2017). Thus, the incidence of metabolic syndrome in patients with depression is relatively high, and conversely, patients with metabolic syndrome are more likely to suffer from depression (Kahl et al., 2015; Ghanei Gheshlagh et al., 2016). The two diseases are likely to be causal to some extent (Toker et al., 2008; Takeuchi et al., 2009; Foley et al., 2010; Lamers et al., 2013; Trief et al., 2014). We showed that although MSG treatment resulted in abnormal lipid metabolism and PH could aggravate lipid deposition, treatment with the antidepressant escitalopram could improve lipid metabolism.

In summary, our results clearly revealed that subtotal hepatectomy aggravated the anxiety-like depressive behaviors changes in the MSG rat model, and this effect was related to pathological changes in the OFC and disorder of neurotransmitters. The degree of hepatic steatosis was also aggravated by PH. The antidepressant escitalopram could alleviate the pathological changes in both OFC and liver in this model. Nevertheless, because depression is a multifactorial condition and closely affected by social events, further studies are needed to examine the efficacy of antidepressants in CLD patients with depression.

## Data Availability Statement

The data used to support the findings of this study are included within the article.

## Ethics Statement

All relevant ethical safeguards have been met in relation to patient or subject protection, or animal experimentation.

## Author Contributions

B-BZ, PW, and HL designed and conceived the study. B-BZ, L-LC, Q-HL, G-JX, BX, and Z-FL carried out the experiments. BZ, LC, and QL wrote the manuscript.

## Conflict of Interest

The authors declare that the research was conducted in the absence of any commercial or financial relationships that could be construed as a potential conflict of interest.
